# Popular media and the bombardment of evolution misconceptions

**DOI:** 10.1186/s12052-022-00179-x

**Published:** 2022-12-16

**Authors:** Daniel G. Ferguson, Jessica Abele, Sarah Palmer, Jordan Willis, Connor McDonald, Chandler Messer, Juliana Lindberg, T. Heath Ogden, Elizabeth G. Bailey, Jamie L. Jensen

**Affiliations:** 1 Department of Biology, Brigham Young University, 4102 LSB, Provo, UT 84602 USA; 2 Department of Biology, Texas State University, San Marcos, TX 78666 USA; 3 Department of Biology, Utah Valley University, Orem, UT 84058 USA

**Keywords:** Popular culture, Social media, Evolution education, Memes, Biology, Preconceptions

## Abstract

**Background:**

Many students enter science classrooms with misconceptions about scientific principles. One of the most perceived controversial scientific principle for students is evolution. Students struggle to learn and accept evolution due to the many misconceptions students have interacted with before they enter a biology class. Evolution misconceptions come from many sources, such as religious beliefs, textbooks, and even unprepared educators. However, with students spending on average over seven hours a day viewing popular media, it is crucial to investigate further the accuracy of the portrayals of evolution in popular media.

**Results:**

We gathered data on the sources students saw evolution portrayed in popular media and determined what misconceptions were present in these popular media references. We found that 96% of the popular media references mentioned by students in our study inaccurately depicted evolution. The two most common misconceptions we observed in popular media were that evolution was depicted as a linear process and that individual organisms evolve instead of populations.

**Conclusion:**

Popular media does a poor job depicting evolution, which may be why many students are hesitant to learn evolution and overcome misconceptions. We suggest that these incorrect portrayals of evolution may provide an engaging way to teach correct evolutionary principles in the classroom.

## Background

Science educators face the constant challenge of students entering classrooms with wrong ideas about scientific principles obtained through life experiences (Driver et al. 1994). Learned belief that is incorrect or contradicts scientific consensus is known as a misconception (Karpudewan et al. 2017). Misconceptions may be informally obtained through life experiences and intuitive theories about how humans understand biological principles (Coley and Tanner 2015; Shtulman 2017). In some cases, it can be reinforced through ineffective teaching strategies used in science classrooms (Gunyou 2015). This has been exceptionally true with the theory of evolution, as many students entering biology classes hold misconceptions about evolution (Bishop and Anderson 1990; Alters and Nelson 2002; Evans and Diamond 2005; Wescott and Cunningham 2005; Yates and Marek 2014). Although evolution is the central explanatory principle in biology (Dobzhansky 1973; Plutzer and Berkman 2008; American Association For The Advancement of Science 2011), it is one of the most misunderstood concepts by the general public and is one of the most widely controversial, polarizing scientific theories portrayed in the media and by the general public (Nadelson and Hardy 2015; Pobiner 2016). The reasons evolutionary theory is misunderstood are still a topic of debate. Still, we know that misconceptions about evolution come from various sources, some of which come from textbooks and unprepared educators (Meadows et al. 2000; Griffith and Brem 2004; Meadows 2009; Glaze et al. 2015; Borgerding 2017; Tolman et al. 2021), while others may come from everyday life or perceived conflict with cultural beliefs (Nelson et al. 2019; Barnes et al. 2020).

## Evolution and common misconceptions

Evolution misconceptions can come from various sources such as social settings, teachers and textbooks, and even religious settings. Evolution is also a complicated topic that can be difficult for many students and the general population to understand fully (Bybee 2001; Rector et al. 2013). One of the difficulties is that words used in science such as “theory,” “fact,” and “proven” are used differently in standard American vernacular than in science, and this can impede how students learn biological principles, like evolution (Evans and Diamond 2005; Glaze et al. 2015; Green and Delgado 2021). For example, in one study, Larochelle and Desautels (Larochelle and Désautels 1991) found that when interviewing students, when the word “theory” was used to explain scientific principles, many students viewed those ideas as one person's opinion instead of a collaboration of scientists working together (Nelson et al. 2019; Larochelle and Désautels 1991). If students believe that evolution is “only a theory,” they will likely feel uncertain about evolutionary theory's veracity (Dagher and Boujaoude 2005). Another common misconception is the image depicting hominids, in a linear process, eventually turning into humans. This image was created by Rudolph Zallinger in 1965 for an article for the *Early Man* Volume of *Life Nature Library* (Krogman 1965) and has been problematic for many students who see it as the way scientists believe evolution happens (Green and Delgado 2021).

Students do not just obtain misconceptions from contradictions in word meaning or common images; they may also acquire misconceptions from the classroom, teachers, and textbooks.

Textbooks are tools used by teachers that help facilitate learning in the classroom (Barrass 1984), but textbooks may also contain misconceptions (Tshuma and Sanders 2015). When reviewing textbooks for grades 10–12, Tshuma and Sanders (Tshuma and Sanders 2015) found 27 different evolution misconceptions, the most common being something to the effect of “individual organisms evolve,” “environmental changes cause evolution,” and “organisms adapt during their lifetimes.” Misconceptions about evolution are found not only within textbooks and curricula but also among teachers (Rutledge and Mitchell 2002), and evolution misconceptions may be created in class (Bohlin et al. 2017; Leeder 2019). If teachers have an incorrect understanding of evolution, they may pass those incorrect ideas down to their students. Teachers can even pass on evolution misconceptions by avoiding teaching evolution, especially macroevolution (i.e., (Padian 2010)), or teaching creationism instead (Plutzer et al. 2020). Teachers can also pass on evolution misconceptions by claiming evolution as the central theme in a biology class and then poorly integrating evolutionary principles within other topics such as genetics (Nehm et al. 2019).

Students seem to be coming across misrepresentations of evolution in various settings; some are educational (as described above), and some are not. For example, evolution is often discussed in religious settings such as churches and Sunday schools, where evolution may be incorrectly described or represented. The misrepresentation of evolution in a religious setting may lead to conflict between religious and secular worldviews. The conflict between religious and secular worldviews may be strengthened if religious students come across a statement from scientists claiming that religion and science are incompatible and even suggest that science can disprove God(s) (Krauss 2015; Coyne 2016) or that evolution is anti-God and that by accepting evolution, Christians are in effect abandoning their faith (Barnes et al. 2021); the idea that evolution and faith are incompatible is a misconception about evolution.

Given all of the above influences on evolution understanding that generally occur in academic and non-academic settings, there is still a gap in our understanding of the influence that non-educational portrayals of evolution may have on students’ perceptions of evolution, specifically, popular media as a source of evolution misconceptions (Brattstrom 1999; Jakobi 2010; Bucklin and Daniel 2017; Asberger et al. 2021). As far as we know, little research has been done on evolution misconceptions in popular media. However, recent studies have highlighted that children’s books depicting evolution may convey misconceptions (Adler et al. 2022). Another study looking at videos created and shared with the intent to educate about evolution on YouTube or Google was also found to harbor some misconceptions (Bohlin et al. 2017). Even those intending to educate about evolution are not always accurate, so it would be beneficial for educators to be aware of what potential misconceptions, if any, are coming from these non-academic sources.

This study defines popular media as video games, movies, television series, and social media (TikTok, Instagram, Facebook, etc.) that students typically use or view outside class. A recent survey showed that teens spend, on average, over seven hours a day viewing popular media (Sense and Census 2019). This can be concerning for educators as students struggle to distinguish between accurate and inaccurate information (Leeder 2019). Inaccurate portrayals of evolution in popular media may affect how students understand evolution or interact with it in the classroom. For example, the video game “Spore” was created in response to a growing interest in using digital game-based learning to aid in teaching evolution (Gee 2003; Schrader and McCreery 2008; Squire 2006; Steinkuehler 2005). Although this video game did seem to aid in learning evolution for some students, it may have promoted more evolution misconceptions instead of a better understanding of evolution for other students (Bean et al. 2010).

In addition to video games, evolution misconceptions bombard students via movies such as *X-Men*, which refers to an evolutionary arms race between mutants and humans, or television series such as *The Big Bang Theory,* which shows the monkey-to-man evolution image in the title sequence. Evolution misconceptions can also be seen on social media when friends or family share a meme with a picture of a monkey saying, “If we came from monkeys, why are there still monkeys?” This can be especially problematic when many people spend their time on social media looking not only for social interactions and entertainment (Whiting and Williams 2013) but also for accurate updates about world politics and scientific discoveries: what they view on social media may influence how individuals view the world.

In many cases, inaccurate information travels faster and reaches more locations than accurate information (Vosoughi et al. 2018); this was particularly true for COVID-19 (Bridgman et al. 2020). Previous research suggests that as students come across inaccurate depictions of science, specifically evolution, they will have difficulty integrating what is taught by their teachers in class (Osborne and Freyberg 1985; Smith and Abell 2008). A previous article highlights the potential for students to be bombarded with evolution misconceptions given the amount of time students spend viewing popular media daily (Ferguson and Jensen, in press) and how this influences their understanding of evolution. Although some studies have mentioned a need to investigate popular media as a potential avenue for evolution misconceptions (Asberger et al. 2021), no studies have examined evolution misconceptions in popular media or tried to understand the effect of inaccurate portrayals of evolution in popular media on students' knowledge of evolution.

This study explored evolution portrayals in popular media as a source for evolution misconceptions. We think it is important to know from where students might obtain evolution misconceptions so that educators can better address them in the classroom (Vaughn 2017). We first investigated where students have seen evolution portrayed in popular media. Then we analyzed those popular media evolution references to determine if they harbored any evolution misconceptions. By analyzing cases where students have seen evolution portrayed in popular media, we can better address evolution misconceptions in the classroom while potentially using the incorrect popular media references as an avenue for student engagement when discussing evolution.

## Methods

### Sample population

To determine if misconceptions about evolution were found in popular media, we surveyed students to identify evolution references they had seen. We recruited student participants from several introductory biology classes at a large private religious university in the western United States (N = 342) and a large public open-enrollment university in the western United States (N = 17) for a total of 359 student respondents. The students who participated in this survey were predominantly traditional first-year students enrolled in an introductory biology course. They took either a general education biology course for non-science majors or an introductory biology course for science majors. In the private religious university, the survey was given as an assignment. The survey was sent out at the public university as an announcement over a learning management system to see if anyone was interested in helping with research; it did not count towards students’ grades or extra credit, hence the low participation rate.

### Survey instruments

#### Popular culture evolution references

In this survey, we wanted to know where students have seen evolution portrayed in popular media. Students were asked, “*Have you ever seen the process of evolution, images of evolution occurring, or any other form of evolution portrayal in any form of popular media (e.g., movies, TV programs, books, video games, internet memes, *etc*.)? In which media sources have you seen this? Mark all that apply.”* For this question, they could select from social media/memes, books/articles/magazines, movies, TV programs, video games, and others. If they indicated others, they could describe their reference in more detail. Students were then asked a series of questions about the specific source, such as: *“What was the specific source (e.g., if it was a book, what was the book? if it was a movie, what was the movie? *etc*.),” “In a few sentences, describe the depiction of evolution,”* and *“In one sentence, explain the intended main idea of the image/video clip/skit/*etc*.”* Students were able to answer these questions up to four times, with four different popular media references. If they did not have any more references to report, they were able to move to the next part of the survey. Students were also explicitly asked about memes they had seen that portrayed evolution; they could optionally upload an example meme.

#### Popular culture coding

Once we received the references from the survey, a group of seven researchers (DGF, JA, SP, JW, CM, JL, CM) reviewed the student-identified media to determine if any evolution misconceptions were portrayed. Only 169 popular media references described were clear enough to be identified by researchers as specific media sources. From these 169 references, we identified 73 unique references; see Tables 1 and 2 for details (we allowed multiple movies within a series such as *Planet of the Apes* or *X-Men* which identified as unique references*)*.

**Table 1:** Popular media that portrayed evolution that was viewed by the researchers and the misconceptions that were identified in the references

Popular media references	Misconceptions
**Books**	
Daddy long legs	5
Dragon slippers	9
Far Side	1, 7
Hound of the far side	4
Series animorphs	1
Goosebumps	1
**Movies**	
The time machine	2
2001: space oddesy	4, 5
X-Men (whole series)	1, 4, 7
The croods	0, 1, 2, 6, 9
Jurassic park (whole series)	0, 1, 2, 3
Hitchhiker’s guide to the galaxy	5
Ice age	3, 4, 7
Evolution	1, 3
Planet of the apes (whole series)	1, 4, 5, 6
Minions	1, 4
High school musical	3
Tarzan	3, 5
**Television**	
Spongebob squarepants	0
The big bang theory	3, 5
Phineas and ferb	1, 3, 5, 6
Pokemon	1, 3, 4
Friends	8
Bill nye the science guy	2, 6
The amazing world of gumball	5
Walking with monsters	3
**Video games**	
Ancestors—the humankind odyssey	6
Pokemon	1, 3, 4
Spore	4

Movies series were combined in the table, but were coded individually. This table does not show what memes were also viewed by the researchers. It is possible for some references to have a code for both a misconception and having no misconception, as some segments may have portrayed evolution accurately while other did not

**Table 2:** The identified misconceptions and codes

Code	Misconceptions
0	Media source contains no misconceptions
1	Individual organisms evolved instead of populations
2	Teleological: organisms intentionally evolve
3	Evolution was depicted or described as a linear process
4	Only the fittest survive. The fittest organisms in a population are those that are strongest, healthiest, fastest, and/or largest
5	Humans directly evolved from apes
6	Adaptation was used as a means trying to evolve
7	Changes in the environment cause mutations
8	There is a conflict between religion and evolution
9	Dinosaurs and humans lived at the same time

Before coding, we created a list of common evolution misconceptions (see Table 3) using the Biological Evolution Literacy Survey (BELS) (Yates and Marek 2014; Yates and Marek 2011) and a guide to teaching evolution from the University of California Museum of Paleontology (Misconceptions about evolution 2021). During the coding, we determined that some misconceptions from our original list were very similar to other misconceptions. In those cases, we combined the categories. In other cases, we identified new misconceptions, in which cases we created new categories.

**Table 3:** Common misconceptions found in the literature

List of evolution misconceptions
Evolution is linear process
Evolution is just a “theory”
Individual organisms evolve
Evolution is a teleological process
Humans evolved directly from apes
Only the strongest organisms evolve
Uses the wrong definition of adaptation
There is no evolution misconception present
Dinosaurs and humans lived at the same time
There is a conflict between religion and evolution
Evolution is caused by the environment (not mutations)
Evolution results in progress; organisms are always getting better through evolution
Evolution is a quick process that makes significant changes to a population over a single generation
Only the fittest survive. The fittest organisms in a population are those that are strongest, healthiest, fastest, and/or largest

To establish inter-rater reliability, we performed ten iterations of individual coding followed by group discussion. To do this, we watched, inspected, or played each popular media reference together as a group, coded them individually, and discussed them as a group (popcorn was provided). If necessary, we re-watched after discussion and coded again. By doing so, we reached a 90% agreement on coding. In all cases when re-watching was necessary, we found that the popular media reference depicted evolution in more than one way and thus fit more than one code. In these cases, we recorded the reference as having more than one misconception. Once we reached 90% interrater reliability, members of the research group were individually assigned the remaining references to code. See Tables 1 and 2 to see what popular media references were viewed and which misconceptions we observed.

## Results

### Where students have seen evolution portrayal in popular media

**Fig. 1:**
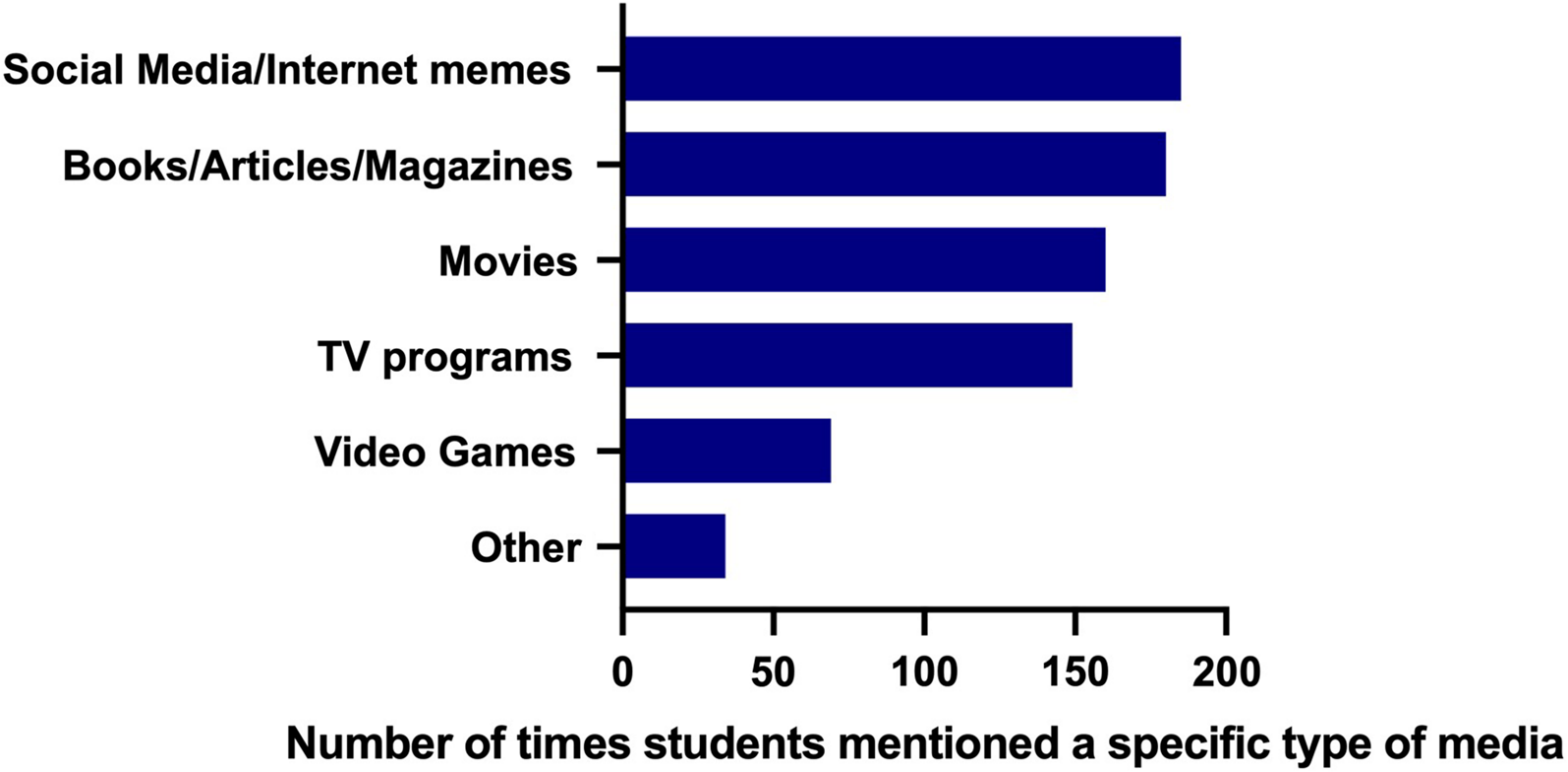
Graph depicting places students mentioned seeing evolution portrayed in popular media (N = 359). Students could select multiple choices

The primary place students saw evolution portrayed in popular media was through social media and internet memes, with 185 students (52%) mentioning social media and memes (see Fig. 1 for details). One hundred eighty students (50%) mentioned seeing evolution portrayed in books, articles, and magazines—most students who mentioned books and magazines referred to biology textbooks (which we did not identify as popular media) or scientific magazines like *National Geographic*. One hundred sixty students (45%) mentioned seeing evolution portrayed in movies and 149 students (42%) saw evolution portrayed in television programs. Whereas 69 students (19%) mentioned evolution being portrayed in video games, and 34 students (9%) mentioned seeing evolution portrayed in other settings such as discussions of evolution among family, friends, or religious gatherings.

### Evolution misconceptions identified in popular media

From the student-mentioned popular media references, we identified eight evolution misconceptions. The most common evolution misconception identified in popular media was *Individual organisms evolved instead of populations*, which was found in 22% of the student-mentioned references. *Evolution being depicted or described as a linear process* and *evolution is teleological* were the second most common evolution misconception we found in popular media; they were observed in 16% of the references. Fourteen percent of the popular media references we viewed depicted the misconception *Only the fittest survive* or *The fittest organisms in a population are those that are strongest, healthiest, fastest, and/or largest*. For details about other misconceptions, we found in popular media, see Table 4. Of the 73 evolution references we viewed in popular media, 98% of them portrayed evolution incorrectly (see Tables 1 and 2 for more details). Only 2% of the student-mentioned evolution references portrayed evolution correctly. Our data support the notion that when students view evolution in popular media, it is usually portrayed inaccurately.

**Table 4:** The percentage of evolution misconceptions observed in popular media (N = 73, with a total of 140 misconceptions)

Misconceptions found in popular media references	Percentage of times observed in a popular media reference (%)
Individual organisms evolved instead of populations^a^	22
Teleological: organisms intentionally evolve^a^	16
Evolution was depicted or described as a linear process	16
Only the fittest survive. The fittest organisms in a population are those that are strongest, healthiest, fastest, and/or largest^a^	14
Humans directly evolved from apes^a^	14
Adaptation was used as a means of trying to evolve	10
Changes in the environment cause mutations	2
There is a conflict between religion and evolution	2
Dinosaur and humans lived at the same time	2

^a^refers to misconceptions found among students in our population

## Discussion

Our study highlights evolution misconceptions found in popular media viewed by college-aged students. Many students mentioned seeing representations of evolution on social media and memes, as well as in movies, television series, and video games. We found that overwhelmingly when evolution is portrayed in popular media, it is portrayed inaccurately. Many of the identified evolution misconceptions found in popular media were also evolution misconceptions held by the students in high school classes and introductory biology classes (Yates and Marek 2014; Nelson et al. 2019; Jakobi 2010; Cunningham and Wescott 2009).

### Students have seen evolution portrayed in popular media

Our first aim was to understand where students have seen evolution portrayed in popular media. We gathered this data by surveying students and asking them where they have seen evolution portrayed in popular media. Most students mentioned seeing evolution portrayed in memes (52%) and books and magazines (50%). Many students who mentioned seeing evolution portrayed in books and magazines specifically mentioned their previous textbooks or a scientific magazine they came across, but some students did mention books that we identified as popular media (e.g., *Animorphs*). We did not specifically look at textbooks in this study, as studies have already identified textbook misconceptions (i.e., (Cunningham and Wescott 2009)). Still, textbooks probably portray evolution more accurately than popular media. Many of the students also mentioned seeing evolution portrayed in movies (45%), TV series (Jakobi 2010), and video games (19%).

We were not surprised that many students mentioned seeing evolution in their biology textbooks; however, it was surprising that more students reported seeing evolution portrayed on social media/memes than in books or textbooks. A previous study found that most ten-year-old students cited their books/magazines as a place to learn about the appearance of a scientist (Tan et al. 2017). In contrast, many students in another study cited movies, TV shows, and video games as places where they have seen a scientist portrayed (Szu et al. 2017). Although the previously mentioned study looked at a younger age group, there seems to be a shift in where individuals obtain scientific information in current generations. For example, 62% of adults view social media as a place to obtain news and gather new information (Pew. 2016). With the amount of time people are spending on social media, understanding how social media influences the knowledge individuals obtain and how that knowledge influences their understanding of correct scientific principles may be interesting for future research moving forward.

### Evolution misconceptions in popular media

The second aim of this research was to understand what misconceptions are present in popular media portrayals. We gathered these data by asking students to give specific examples of where they have seen evolution portrayed in popular media, upload memes portraying evolution, and then determine which misconceptions were found in these references. Of the 73 references we viewed, 98% of them portrayed evolution incorrectly, meaning only 2% of the observed references portrayed evolution accurately.

The most common misconception we observed in popular media was that *Individual organisms evolve instead of populations* which we observed in 22% of the references. An example of this misconception in popular media can be observed in the popular video games and television series *Pokémon*. In *Pokémon*, trainers can capture and collect creatures called Pokémon and have them battle other trainers’ Pokémon or wild Pokémon. During these battles, if Pokémon win, they gain experience. If they gain enough experience, they sometimes go through what is described in *Pokémon* as “evolution,” a process where an individual Pokémon instantly changes from one form to another (although some Pokémon need specific stones or items to evolve). During these events, Pokémon can grow stronger, gain new limbs, lose limbs, grow larger, and sometimes gain new battling abilities. Each Pokémon has a set linear evolution path; for example, a fire-type Pokémon named Charmander evolves into Charmeleon (a bigger, stronger form) after gaining enough experience. After gaining more experience, Charmeleon will evolve into Charizard (an even bigger form that obtains wings), the final evolutionary form of Charmander. Within the game and TV series *Pokémon*, evolution is mislabeled as a quick process that happens instantly to an individual organism instead of a slow process at a population level, which is more akin to the biological concept of metamorphosis than evolution. It also depicts evolution as a process that makes organisms bigger, better, faster, and stronger. This idea about evolution is also incorrect, as evolution does not have an endpoint and does not necessarily mean organisms have to grow stronger or bigger to survive and pass on their genes. In *Pokémon,* we observed the misconception that *Individual organisms evolve instead of populations,* but also two other evolution misconceptions: *Quick evolution* and *Evolution makes things stronger*.

However, the second most common evolution misconception found in popular media was the misconception that *Evolution was depicted as a linear process*, which we observed in 16% of the references. Usually, this was shown as a modification of the image, “March of Progress” image (e.g., (Brattstrom 1999; Cunningham and Wescott 2009; Szu et al. 2017; Pew. 2016)). For example, in the movie *Ice Age,* there is a scene where a group of characters is walking through an ice cave when one of them (“Sid the sloth”) comes across a group of organisms that seem to be frozen in a linear fashion. The image suggests that the first organism turned into the next, and the next turned into a different organism until an organism turned into Sid, the sloth. This example of evolution portrayed in popular media depicts evolution as happening in a linear fashion instead of branching events and was one of the most common evolution misconception we observed in the popular media references we viewed and watched.

Some studies have shown that popular media influences how the public perceives science and scientists (Brattstrom 1999; Tan et al. 2017; Pew. 2016; Ross et al. 2013). This may also be the case with popular media's influence on evolution understanding and acceptance, especially as we saw that 98% of the evolution references we viewed were inaccurate. Still, more research is needed to address the effects of popular media on evolution misconceptions. It may well be that these misconceptions propagated by popular media strongly influence student acceptance of evolution and evolution understanding.

### Future directions of popular media in the classroom

Although it seems that popular media may inaccurately portray evolution, there may be some benefits to using these incorrect representations in the classroom to teach correct principles. For example, Van Riper (Riper 2003) argues that even with popular media getting ideas about science incorrect, these incorrect portrayals of science and evolution can lead to great classroom discussions and teachable moments. One other study claimed that the video game *Spore* (which was created as a means to teach evolution) solidified evolution misconceptions in students unless playing the game was followed with pedagogical support, which seemed to aid in decreasing evolution misconceptions and strengthening evolution learning (Bean et al. 2010). Although popular media often gets it wrong, there may be moments where educators can show inaccurate portrayals of evolution in the class to help engage students in their learning and help correct inaccurate misrepresentations of evolution. But there are no studies that specifically look at the effect of such an intervention; moving forward, this is something we would like to pursue. An example of this might be something as simple as teachers showing popular media clips depicting evolution in class and letting their students discuss these clips individually or as a group. The students could discuss how the popular media reference depicts evolution and decide if it is an accurate depiction of evolution or not.

## Limitations

This article looks at how evolution is portrayed by popular media. One of the central weaknesses of this study is that the references we viewed were not very diverse in that they were identified from a highly homogenous population of students with similar religious views. Many students in this population have strict media habits regarding what they view (e.g., many do not view movies or shows with R or Mature ratings), so our observed references may not be a diverse sample of what evolution portrayals are present in popular media. Still, future studies may identify a broader scope of media by using a more diverse sample. We were also unable to look at the effects of popular media on students’ perceptions of viewing a meme portraying evolution versus a book or television series this is a interesting question that would require further investigation as how a student portrays or believes what they see in a specific type of media is currently lacking. Another weakness of this study is that many popular media coders were undergraduate researchers. Most were Seniors who had taken a higher-level evolution course, whereas we also had two Juniors who had not yet taken an evolution course. It is possible that because of a lack of evolution knowledge, we may not have properly identified the misconceptions. However, we believe the popular media references still held evolution misconceptions. We tried to limit the inaccuracies by having discussions about evolution throughout the coding process with popular media references that were ambiguous or confusing. In future studies, it may be helpful to ensure that student researchers get a crash course on evolution before the coding process begins. One other thing to keep in mind is that many of the popular media references we observed were TV series. Due to time constraints, we were not able to watch a whole series, but if the students gave a specific episode or segment, we watched that episode and identified if there were misconceptions.

## Conclusion

This study documented evolution misconceptions in popular media; we observed eight different evolution misconceptions and found that 98% of the popular media references we viewed incorrectly portrayed evolution. We think that using these incorrect popular media representations of evolution in the classroom may be an excellent way to engage students in learning evolution. Although we still lack a solid understanding of how popular media influences students' perceptions and understanding of evolution, we think this research can have a broader impact on how we teach evolution in the classroom. We hope that, if nothing else, educators recognize that students are probably entering their classrooms with misunderstandings of evolution coming from many different sources and may have rarely seen evolution correctly portrayed in popular media.

## Data Availability

Data will be freely available.
